# Host transcriptional responses in nasal swabs identify potential SARS-CoV-2 infection in PCR negative patients

**DOI:** 10.1016/j.isci.2022.105310

**Published:** 2022-10-07

**Authors:** Amanda M. Saravia-Butler, Jonathan C. Schisler, Deanne Taylor, Afshin Beheshti, Dan Butler, Cem Meydan, Jonathon Foox, Kyle Hernandez, Chris Mozsary, Christopher E. Mason, Robert Meller

**Affiliations:** 1 KBR, Space Biosciences Division, NASA Ames Research Center, Moffett Field, CA 94035, USA; 2 NASA Ames Research Center, Moffett Field, CA 94035, USA; 3 COVID-19 International Research Team, Medford, MA, USA; 4 McAllister Heart Institute, Department of Pharmacology, and Department of Pathology and Lab Medicine, The University of North Carolina at Chapel Hill, Chapel Hill, NC 27599, USA; 5 Department of Biomedical and Health Informatics, The Children’s Hospital of Philadelphia, Philadelphia, PA 19104, USA; 6 Department of Pediatrics, Perelman School of Medicine, University of Pennsylvania, Philadelphia, PA 19104, USA; 7 Stanley Center for Psychiatric Research, Broad Institute of MIT and Harvard, Cambridge, MA 02142, USA; 8 Department of Physiology, Biophysics and Systems Biology, Weill Cornell Medicine, New York, NY, USA; 9 The HRH Prince Alwaleed Bin Talal Bin Abdulaziz Alsaud Institute for Computational Biomedicine, Weill Cornell Medicine, New York, NY, USA; 10 Department of Medicine, University of Chicago, Chicago, IL, USA; 11 Center for Translational Data Science, University of Chicago, Chicago, IL, USA; 12 New York Genome Center, New York, NY, USA; 13 The Feil Family Brain and Mind Research Institute, Weill Cornell Medicine, New York, NY, USA; 14 The WorldQuant Initiative for Quantitative Prediction, Weill Cornell Medicine, New York, NY, USA; 15 Neuroscience Institute, Department of Neurobiology/ Department of Pharmacology and Toxicology; Morehouse School of Medicine, Atlanta, GA 30310, USA

**Keywords:** Biological sciences, Immunology, Virology, Transcriptomics

## Abstract

We analyzed RNA sequencing data from nasal swabs used for SARS-CoV-2 testing. 13% of 317 PCR-negative samples contained over 100 reads aligned to multiple regions of the SARS-CoV-2 genome. Differential gene expression analysis compares the host gene expression in potential false-negative (FN: PCR negative, sequencing positive) samples to subjects with multiple SARS-CoV-2 viral loads. The host transcriptional response in FN samples was distinct from true negative samples (PCR & sequencing negative) and similar to low viral load samples. Gene Ontology analysis shows viral load-dependent changes in gene expression are functionally distinct; 23 common pathways include responses to viral infections and associated immune responses. GO analysis reveals FN samples had a high overlap with high viral load samples. Deconvolution of RNA-seq data shows similar cell content across viral loads. Hence, transcriptome analysis of nasal swabs provides an additional level of identifying SARS-CoV-2 infection.

## Highlights

• Nasal swab RNA analysis identifies SARS-CoV-2 infection in PCR-negative samples
• Host responses to SARS-CoV-2 infection are biphasic
• GO analysis identifies common biological responses regardless of SARS-CoV-2 viral load

## Graphical abstract

**Figure:**
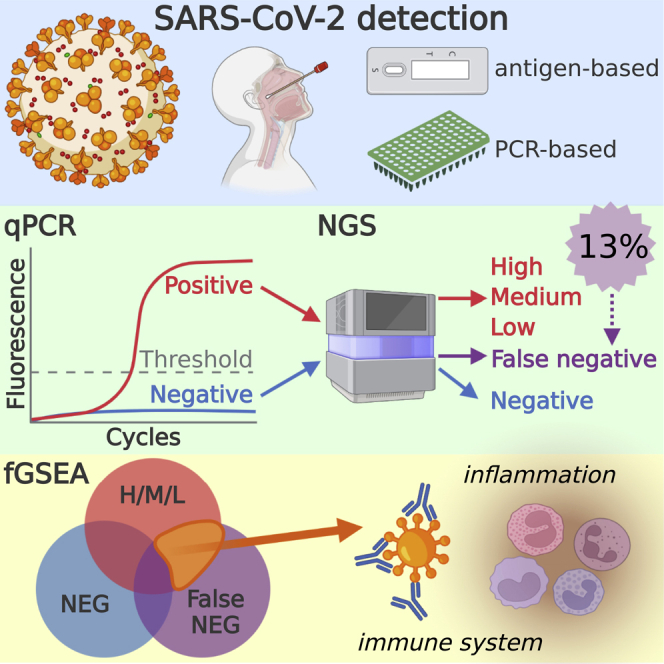

## Introduction

The SARS-CoV-2 pandemic continues to disrupt everyday life, with over 296,496,809 confirmed cases and over five million deaths (WHO 07-JAN-2022) (Organization, 2020). One of the initial responses in the biomedical field was the development of diagnostic tests for the rapid and sensitive detection of SARS-CoV-2 infection, resulting in various molecular PCR and antigen-based tests (Islam and Iqbal, 2020; Liu et al., 2020; Okamaoto et al., 2020). Many of these tests continue to operate under Emergency Use Authority regulations from the FDA in the US. However, many anecdotal reports of people with COVID-19 symptoms with negative tests are common. Here, we investigated the host transcriptional response of potential PCR false-negative subjects using RNAseq data from a shotgun transcriptome sequencing study (Butler et al., 2021). Indeed, multiple studies from biospecimens show activation of immune responses (alpha and gamma interferon responses) and bioenergetic responses to (Okamaoto et al., 2020) SARS-CoV-2 infection, as well as viral load-dependent changes in the host transcriptome (Butler et al., 2021; Xiong et al., 2020; Sajuthi et al., 2020). Studies of SARS-CoV-2 and other viruses suggest that the host immune response to a virus may be sufficient to assist in the diagnosis of an infection (Zhang et al., 2021). We used published sequencing data from clinical specimens obtained from 670 patients tested for SARS-CoV-2 in the New York area in early 2020 (first wave), resulting in 192 positive and 389 negative test results via quantitative PCR assay (phs002258.v1.p1). Here, we show evidence for false-negative SARS-CoV-2 detection in 42 patients based on RNA-sequencing coverage of the SARS-CoV-2 genome and the host transcriptome.

## Results

### Identifying potential false-positive SARS-CoV-2 infections using RNA-seq

**Figure 1:**
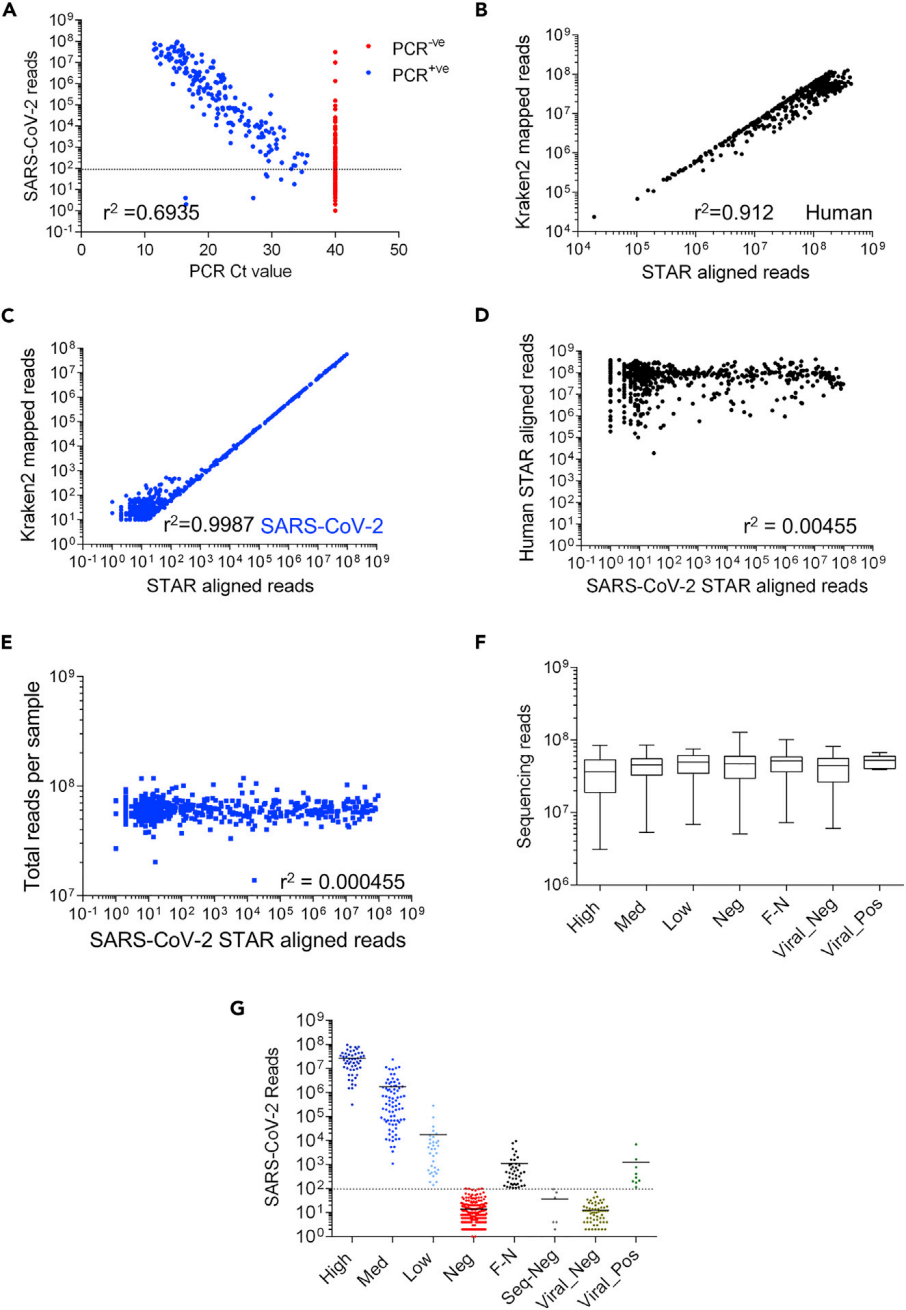
Comparison of Human and SARS-Cov-2 aligned reads from nasal pharyngeal swabs (A) Comparison of PCR Spike gene Ct values for samples vs STAR SARS-CoV-2 aligned reads. A PCR Ct value of 40 was defined as PCR negative (PCR -ve: red, 474 samples) and a Ct value below 40 was defined as positive (PCR + ve: blue, 194 samples). The dotted line denotes a sequencing cut off value of 100 reads. Pearson correlation of PCR-positive samples (Ct values vs log10 number of Sequencing reads) yields a slope of −3.7 and r^2^ value of 0.6935. (B) Comparison of Kraken2 and STAR aligned reads to the human reference genome (GRCh38). Pearson correlation of log10 transformed kraken vs STAR aligned reads to GRCh38 yields a slope of 0.89 and r^2^ value of 0.912. (C) Comparison of Kraken2 and STAR aligned reads to the SARS-CoV-2 reference genome (Wuhan strain). Pearson correlation of log10 transformed kraken vs STAR aligned reads to SARS-CoV-2 genome yields a slope of 0.88 and r^2^ value of 0.978. (D) Comparison of STAR aligned reads to the SARS-CoV-2 vs. human reference Genome (GRCh38) each point represents one sample. Pearson correlation of log10 transformed data yields a slope of 0.019 and r^2^ value of 0.00455. (E) Comparison of STAR aligned reads to the SARS-CoV-2 reference genome vs. the total number of reads in the sample (sequencing depth). Pearson correlation of log10 transformed data yields a slope of 0.0008 and r^2^ value of 0.000455 (F). Total number of reads obtained from all samples split by SARS-CoV-2 status based on PCR and Sequencing results. There were no significant differences between sample read number across categories (1-way ANOVA with post hoc Bonferroni’s test). Data shown are median (min to max values) of 52, 81, 33, 275, 42, 66 & 9 samples, respectively. (G) Separation of samples into categories by PCR (High: Ct ≤ 18, Med: 18 < Ct ≤ 24, Low: 24 < Ct < 40, Neg: Ct ≥ 40) and Kraken2 (Viral) values. We used a total aligned reads values of 100 as our cutoff for positive / negative identification of SARS-CoV-2 by sequencing (dotted line). Samples that were PCR negative and sequencing positive were designated as false negative (F–N) samples. Group names indicate SARS-CoV-2 PCR status. (Of note 40 samples had zero values and are not shown). Samples with other viral infections, based on Kraken2 identification of other respiratory virus infection were removed from subsequent analysis. Data shown are individual values of 52, 81, 33, 275, 42, 66, & 9 samples, respectively (see Figure S1A).

Subjects at New York Presbyterian Hospital-Weill Cornell Medical Center were clinically assessed for SARS-CoV-2 infection using a quantitative PCR (qPCR) test. The qPCR test included an amplicon for the E (envelope) gene to detect B lineage beta-coronaviruses and a second amplicon for the S (spike) gene that uniquely detects SARS-CoV-2 (Butler et al., 2021). The qPCR cycle threshold (Ct) value of the S amplicon is inversely related to the sample's amount of starting viral material, with a limit of detection cutoff at Ct ≥ 40. Using RNA-seq data generated from the same nasal swabs, we measured the association between RNA-seq reads aligned to the SARS-CoV-2 genome and Ct values (Figure 1A). We found an inverse linear relationship, r^2^ = 0.69, between Ct values and SARS-CoV-2 reads in subjects deemed positive by PCR (166). In contrast, of the 317 PCR-negative subjects, Ct ≥ 40, we found 42 subjects with over 100 SARS-CoV-2 aligned reads that represent potential false negatives.

We checked the mapping of our data by comparing the relative STAR aligned counts to the human and SARS-CoV-2 genome with a parallel analysis using Kraken2. We observed a similar correlation of reads mapping to both human (Figure 1B) and SARS-CoV-2 (Figure 1C) genomes using both the STAR alignment and Kraken2 tools (Pearson correlation of raw data r^2^ = 0.49 and 0.99, respectively). There was no correlation between the number of SARS-CoV-2 aligned reads and human aligned reads (Figure 1D) or the total number of sample reads (Figure 1E and 1F). The number of gene counts aligned to the SARS-CoV-2 genome was proportional to the number of gene counts as a fraction of the total generated RNA-seq reads (data not shown).

We then classified the subjects into viral load groups based on qPCR Ct and the number of SARS-CoV-2 reads via RNA-seq (Table 1). An additional six samples were found to be false positives and 72 samples tested positive for other respiratory virus based on Kraken2 analysis; we removed these samples from subsequent analyses (Figure 1G).

**Table 1:** Subjects were grouped based on qPCR range, and subjects with a negative qPCR test (Ct = 40) were further divided into false-negatives (SARS-CoV-2 reads >100) or true negatives (SARS-CoV-2 reads <100)

Viral load group	qPCR Ct range	SARS-CoV-2 reads	n
High	≤18	2.69 × 10^7^ ± 3.06 × 10^7^	52
Med	(18–24]	1.73 × 10^6^ ± 1.64 × 10^6^	81
Low	(24–40)	1.74 × 10^4^ ± 9.79 x10^3^	33
False-negative	≥40	1.10 × 10^3^ ± 1.1 x10^3^	42
Negative	≥40	1.38 × 10^1^ ± 1.38 x10^1^	275

SARS-CoV-2 reads are represented by the mean ± interquartile range of n observations.

**Figure 2:**
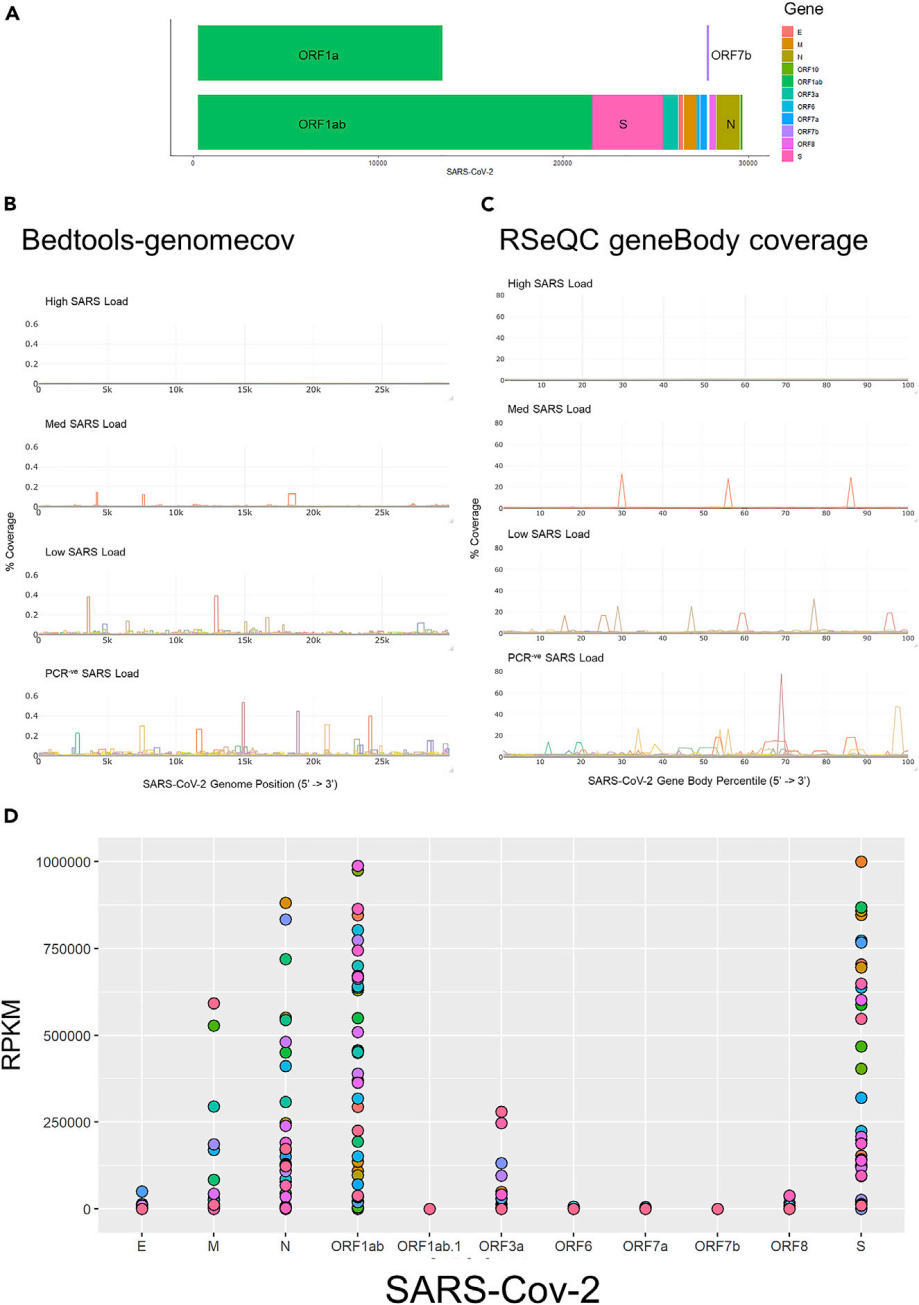
Analysis of alignment of data to SARS-CoV-2 genome (A) Schematic overview of the SARS-CoV-2 genome. (B) Comparison of SARS-CoV-2 genome coverage as determined using Bedtools genomecov. Data were partitioned based on viral load of PCR-positive samples (High, Med, Low), and the PCR negative sequencing positive samples (PCR^-ve^). % Coverage was calculated for each sample as ((number reads in SARS-CoV-2 genome position) / (total number of SARS-CoV-2 aligned reads))∗100. (C) Comparison of SARS-CoV-2 transcript coverage as determined using RSeQC geneBody coverage. Data were partitioned based on viral load of PCR-positive samples (High, Med, Low), and the PCR negative sequencing positive samples (PCR^-ve^). % Coverage was calculated for each sample as ((number reads in SARS-CoV-2 gene body percentile) / (total number of SARS-CoV-2 aligned reads))∗100. (D) Expression of SARS-CoV-2 genes in RNA sequencing data. Read counts were converted to TPM and plotted for each PCR negative sequencing positive sample.

Next, we used Bedtools genomecov to investigate the relative coverage of the SARS-CoV-2 genome (Figure 2A) in each group. In high and medium viral load samples, we observed a consistent 5’ to 3’ coverage of the SARS-CoV-2 genome, whereas in low viral load samples, we observed more coverage variation (Figure 2B). For the false-positive samples, we observed variable coverage of the genome (only 7 samples had high enough coverage for strain analysis). We also investigated the relative coverage of the SARS-CoV-2 transcripts in each group using RSeQC geneBody coverage. In high and medium viral load samples, we observed a consistent 5’ to 3’ coverage of SARS-CoV-2 transcripts, whereas in low viral load samples, we observed more coverage variation (Figure 2C). Finally, for the false-positive samples, we observed variable coverage of the transcripts. The low fragmented coverage of false-positive samples is suggestive of low viral load levels or potentially viral fragments indicative of potential post-infection shedding.

Next, we focused on the 42 false-negative samples and analyzed the number of SARS-CoV-2 aligned transcripts for each annotated SARS-CoV-2 gene. We detected only a few reads aligning to Orf1ab.1, Orf6, and Orf7b; in contrast, we observe a range of expression values of E, M, N Orf10, Orf1ab, Orf3a, and S genes. In the majority of the false-negative samples, the relative abundance of the highest expressed viral genes varied within each sample (Figure 2D). Together, our analyses clearly identified viral RNA from multiple genomic regions of SARS-CoV-2 in 42 subjects, who were determined uninfected by PCR.

### Viral clade analysis

We identified 42 potential false-negative patient samples. From these, we were able to assemble genomes for strain analysis from seven samples (Butler et al., 2021) which were submitted to GISAID. Nextclade analysis identified 19B (1), 20B (1), and 20C (5) strains of the SARS-CoV-2 virus. Compared to the PCR-positive samples (148), we observe relatively higher numbers of 19B and 20B and a similar proportion of 20C strains (71% vs 76%); however, the proportions are likely influence by the low number of samples passing QC (Table 2). The other false-negative samples did not pass QC for genome analysis (data not shown). Due to the low numbers, we were unable to determine whether the strain of virus had an impact on the ability of PCR to detect its presence or not.

**Table 2:** Nextstrain clade analysis of PCR positive and negative samples that passed QC for genome analysis and submitted to GISAID

*PCR status*	Nextstrain clade					*Total*
	*19A*	*19B*	*20A*	*20B*	*20C*	
Negative		1 (14.3%)		1 (14.3%)	5 (71.4%)	7
Positive	9 (6.1%)	8 (5.4%)	16 (10.8%)	2 (1.4%)	113 (76.4%)	148
Total	9	9	16	3	118	155

Data shown are numbers of samples (percent).

Directional response of host gene expression identified viral load-dependent signatures and similarities between low viral load and false-negative subjects

**Figure 3:**
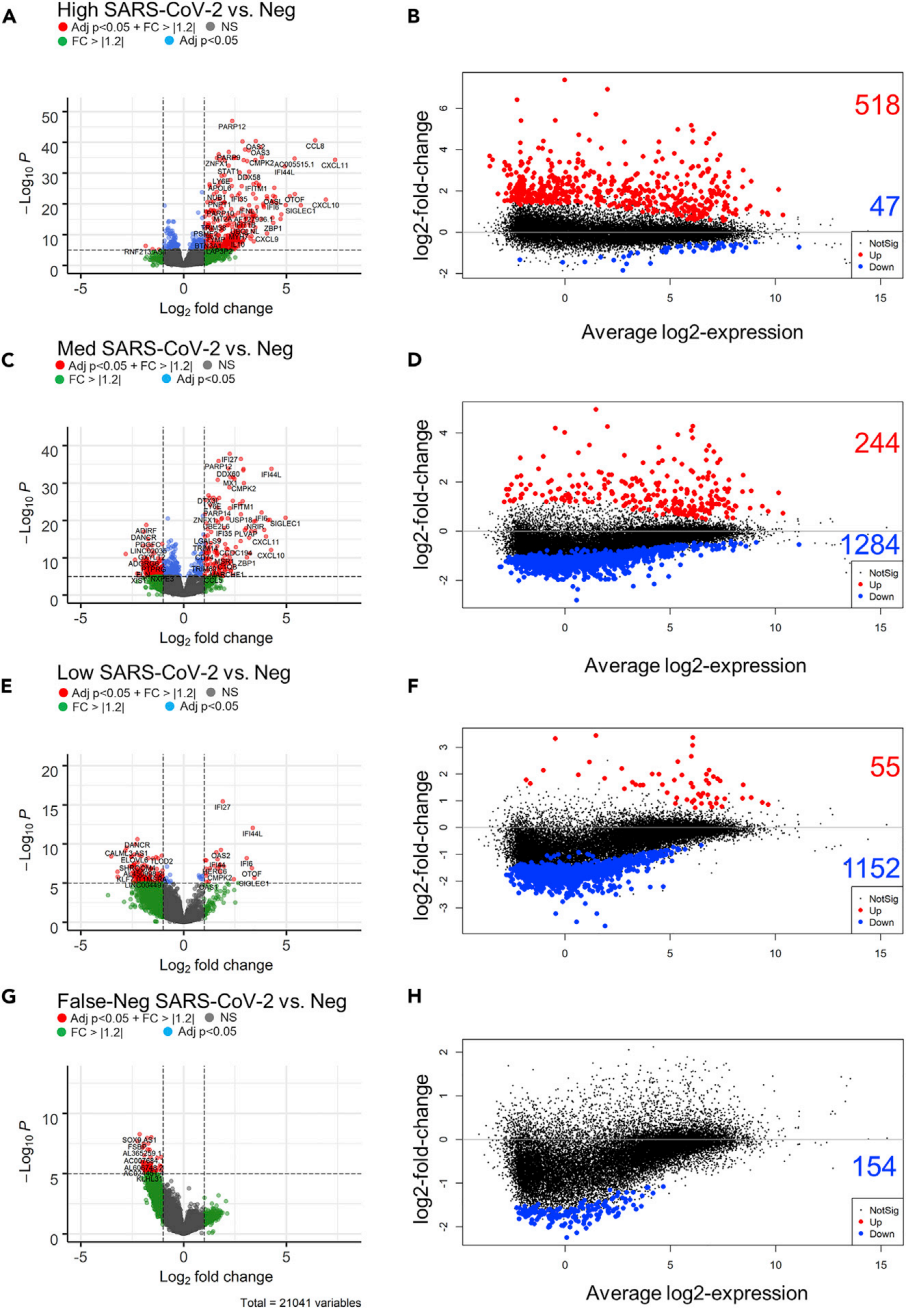
Gene expression in nasal pharyngeal swabs from patients exposed to SARS-CoV-2 based on high, medium, low and (F–N) viral load vs. uninfected patients (PCR and sequence negative samples, Neg) (A, C, E and G) Expression depicted in volcano plots. Volcano plots show the raw unadjusted p value, and the values that pass our inclusion criteria are depicted in red (Differentially expressed genes (log2 fold change > 1.2 or < −1.2 and adjusted p < 0.05 (FDR)). The dotted lines show the cut off raw p (adjusted p value = 0.05 unadjusted p of approximately 1 × 10^−5^) and log2-fold change (±1.2) values. (B, D, F and H) MA plots showing average log2 expression vs. log2-fold change of up (red) and down (blue) regulated genes. Non-significant changes in expression are shown in black. Note the shift from predominantly increased gene expression in high viral load patients, to decreased gene expression in low viral load patients.

**Figure 4:**
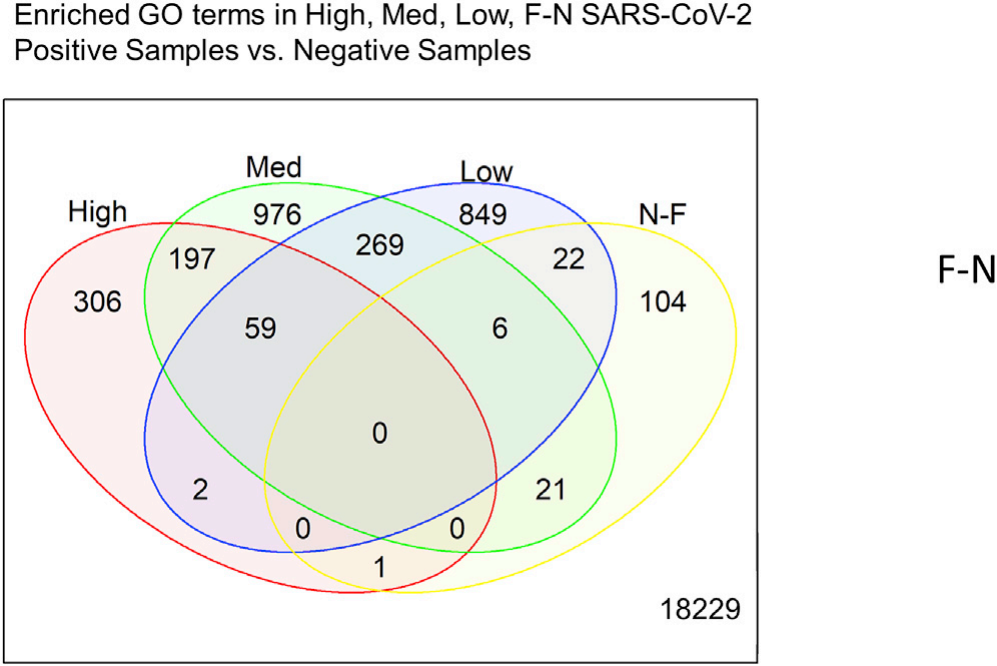
Analysis of genes showing differential expression with varying SARS-CoV-2 viral load The names of significant differentially expressed genes (±1.2 fold change and adjusted p < 0.05 (FDR)) in High, Medium, Low, and F-N samples vs Negative samples). Data were compared using the vennDiagram function of LIMMA. A total of 21, 041 genes were evaluated. Data does not indicate the direction of the change.

We hypothesized that false-negative samples likely reflect a low viral load condition. To test our hypothesis, we used the nasal swab RNAseq data to both profile the host gene expression response and compare the response across our subject groups (Table 1). We defined differential gene expression as an absolute fold change > 1.2 with a false discovery rate <0.05 using negative samples (both by qPCR and RNAseq) as the comparator group (Ritchie et al., 2015; Law et al., 2014). In the host RNA from high viral load subjects, we observed a skewed signature of differential gene expression with over 10-fold more upregulated to downregulated genes (518 up / 47 down, Figures 3A and 3B). This pattern was reversed in both medium viral load, low viral load, and false-negative subjects, 244 up / 1284 down, 55 up / 1152 down, 0 up / 154 down, respectively (Figures 3C–3H). Next, we compared the commonality of the differentially expressed genes and found that false-negative samples had the most overlap with low viral load subjects (28 genes), followed by medium viral load (27 genes), and only one gene in common with high viral load subjects (Figure 4). Among the PCR-positive groups, 59 genes were commonly regulated across viral loads, 197 genes were shared between high and medium viral load, whereas 275 were shared between medium and low viral load. In contrast, only two genes were uniquely shared between high and low viral load (Figure 4). These data suggest that subjects with a high viral load, as determined by nasal swab qPCR, have a distinct directional host transcriptional response compared to other SARS-CoV-2-positive subjects.

### Functional analysis of host gene expression identified host immune and inflammatory responses in false-negative samples

Using our viral load classification, we found the directionality and uniqueness of host expression changes at the individual gene level (Figures 3 and 4). Functional analysis of RNAseq data leverages collections of genes that are related to common pathways, functions, and cellular localizations to identify specific gene sets that are enriched across the transcriptome. We hypothesized that functional analysis of differential gene expression would identify host responses common to SARS-CoV-2 infection as well as host pathways sensitive to viral load. We used fGSEA and topGO to compare the differential gene expression patterns at gene ontology (GO) level.

**Figure 5:**
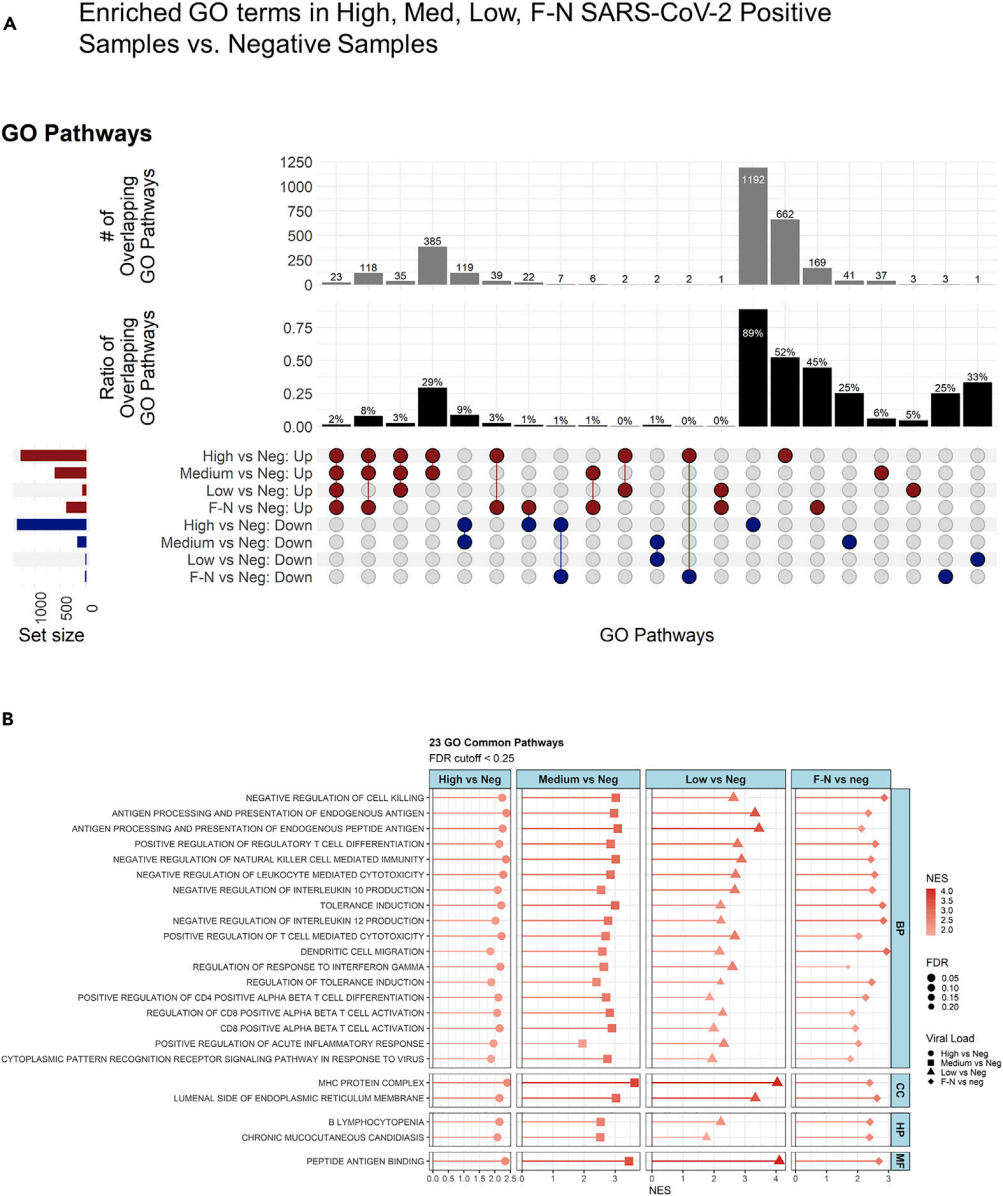
Analysis of gene ontology of samples with varying viral load (A) Lists of differentially expressed genes were analyzed using fGSEA to identify enriched GO pathways. Upset plot for enriched GO terms/ pathways show terms up or down regulated (based on NES) using the R package complex-upset. The cutoff of the pathways are FDR < 0.25. (B) Common pathways (23) represented using lollipop plots.

Our fGSEA analysis (Figure 5A) found that the high viral titer group had the largest enrichment of GO terms, 2,606. The medium titer group had 766 enriched GO terms, 66% of which overlapped with the high titer group. The low titer and false-negative groups had the fewest number of enriched GO terms, 66 and 390, respectively. We identified 23 common GO pathways enriched across all subjects with detectable SARS-CoV-2, via RNAseq, compared to negative subjects (Figure 5A). Remarkably, the 23 common pathways were all upregulated compared to the negative controls and shared inflammatory and immune response pathways observed in viral infections, including in patients with COVID-19 (Figure 5B) (Butler et al., 2021; Xiong et al., 2020; Sajuthi et al., 2020). In addition to the 23 common pathways, there were 188 common pathways between high load, medium load, and false-negative subjects. Surprisingly, there were only six common pathways enriched in both the false-negative and low viral load groups.

### Viral load linked to unique functional host responses at the transcriptional level

Differences in viral load may reflect the infection time course, the effectiveness of host response, or the effectiveness of treatments. Regardless, it is clear from both linear (PCA) and non-linear (tSNE) dimensionality reduction of the host RNAseq data (Figure S2) that the high load samples are the most homogeneous compared to the other groups. As expected, the fGSEA analysis of high viral load samples identified pathways associated with defense responses to viral infections, but also protein targeting to membranes, and mRNA catabolic processes. We also used topGO, a similar enrichment analysis of GO terms, that considers the hierarchical structure of ontologies to increase accuracy. For high viral load samples, topGO analysis shows enrichment of pathways associated with sensory perception signaling pathways followed by RNA and DNA processing (supplementary datas). Our analysis of medium and low viral responses using topGO revealed similar sensory perception and RNA silencing pathways.

Next, we investigated the chromosomal enrichment of genes in the false-negative (PCR negative-sequence positive) samples and observed two significant enrichments sites on chr1p21 and chr8q21, which were also observed in the medium dataset, but not the high dataset (supplementary datas). Gene set enrichment analysis with topGO again reveals enrichment in sensory perception pathways and RNA/ DNA regulatory mechanisms in PCR negative-sequence positive samples (supplementary datas).

### Cell population mixtures were consistent across nasal swab samples

**Figure 6:**
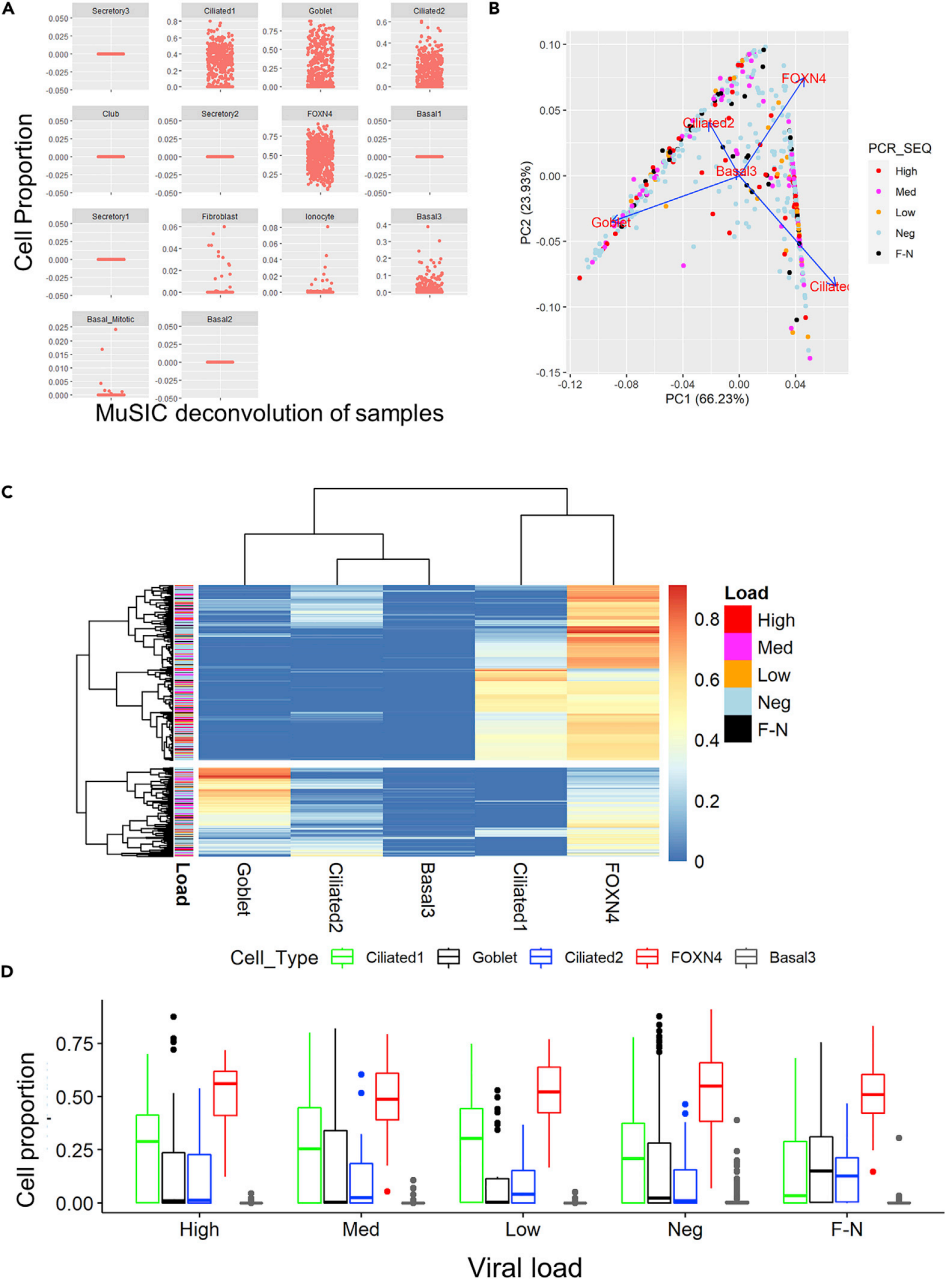
MuSiC deconvolution of RNA-Seq data does not show differential cell populations in different viral load samples Sequence data were deconvolved comparing to a nasal brush and upper airway and lung cell single cell reference data set (Lukassen et al., 2020). (A) Identification of cell proportion in samples based on deconvolution of data. (B) Principal component analysis of cell proportion data as denoted by PCR viral load and sequence identification of SARS-CoV-2 infection (SARS-CoV-2 Viral Load) status of samples. (C) Heatmap of cell proportions (x-axis) by sample ID (y axis). The color denotes the relative proportion of the different cell types in the samples. The color bar by the samples denotes their viral load status. (D) Box plot of airway cell proportion by sample type. No sample showed significant enrichment (2-way ANOVA). Data shown are median +/− quartiles of cell proportions of high (52), med (81) low (33) Neg (275) and FN (42) samples.

Differences in cell populations can influence differential gene expression profiles in biosamples (Bruning et al., 2016). Given the differential gene expression profiles and the results of our functional analyses, we used the RNA-Seq deconvolution tool MuSiC (Wang et al., 2019) and a single-cell airway dataset (Lukassen et al., 2020) to deconvolve our data to predict cell type proportions. We identified cilliated1, cilliated2, goblet, FoxN4, and basal3 cell types as the largest contributors (Figure 6A). We analyzed the cell populations across all samples using principal component analysis (Figure 6B), hierarchical clustering (Figure 6C), and tSNE (Figure S2). Neither method revealed patterns of cell proportions that correspond to viral load status. Finally, we used 2-way ANOVA with cell type and viral load as main factors and did not observe any interaction between cell type and viral load (interaction between PCR status and cell type = 0.06, no group show significant interaction, p = 1.0: post-hoc Tukey test: Figure 6D and S1). These data suggest differences in cell type populations do not explain the differential gene expression we observed.

## Discussion

Analysis of RNA sequencing data obtained from nasal-pharyngeal samples collected during SARS-CoV-2 testing in the New York Region revealed 42 out of 317 (13%) PCR-negative samples had detectable SARS-CoV-2 genomic material, suggesting they were false negatives (F-N) (Figure 1). RNA sequencing data from these potential F-N samples aligned to multiple SARS-CoV-2 genes across the SARS-CoV-2 genome (Figure 2B), suggesting this was not just a single region being detected (erroneously). Gene expression analysis of F-N samples shows a downregulation of gene expression response that was similar to the response of patients with low and medium viral loads (Figure 3), although the genes were different between groups (Figure 4). Gene Ontology analysis showed similar biological pathways are regulated by F-N samples and all SARS-CoV-2 positive patients (Figure 5). Finally, the cellular content of the swabbed samples (as determined by deconvolution) was not different between false-positive samples and other SARS-CoV-2-positive samples (Figure 6). Together, these data support our observation that 13% of PCR-negative samples were false negatives.

Patients showed different host transcriptome responses depending on viral load (Figure 3). Overall responses changed from increased gene expression to decreased gene expression as the viral load reduced (Figure 3). The host response in the F-N samples was most similar to the Med and Low viral response groups (downregulation). However, there were few common regulated genes overlapping between all three groups (Figure 4); out of over 1,000 genes regulated in Med and Low samples, only 22 were common to Med and F-N samples and only 21 were common to Low and F-N samples. Despite few genes overlapping between samples of varying vial load, the biology of regulated genes appears similar among samples (Figure 5). Using both FGSEA and TopGO tools (supplementary datas), we see a concordance of biological pathways regulated by all viral conditions. Interestingly, we see the highest (2.4% of all terms from all samples) overlap with the high viral load group (High, 70), followed by the medium and low viral load group (Figure 5A). This may suggest a biphasic gene response to viral load, or that different genes converge to regulate the same biological pathways.

Different cell proportions may influence gene expression profiles from bulk tissues (Bruning et al., 2016). Deconvolution of RNA seq data using MuSiC did not identify any effects of viral status on the cell proportions of the samples (Figure 6). Attempts to predict the viral status of F-N samples using Random Forest and Neural Network models of top expressed genes were unsuccessful (best models were 5/42 called, data not shown, script provided). However, this may not be that surprising given the variability of gene expression in the samples using PCA and tSNE, even following filtering for the top 5, 10, and 100 most significant DEGs (based on LIMMA adj. p values) between groups does not cleanly resolve the viral status samples (High, Med, Low, F-N from Negative sequencing samples; Figure S2). Together, these data suggest that SARS-CoV-2 infection can be identified by host tissue responses to infection, and that gene expression patterns regulate common biological response pathways to viral infection, but these responses are not due to cell composition of the samples.

The rapid development of PCR-based testing for SARS-CoV-2 contributed to helping monitor and control the disease (Islam and Iqbal, 2020; Liu et al., 2020; Okamaoto et al., 2020). PCR is commonly referred to as the gold standard for SARS-CoV-2 testing (Brooks and Das, 2020; Drame et al., 2020). Any shortcomings of PCR testing are usually limited to failure to detect past infection (Yong et al., 2020), although concerns about the accuracy of early PCR tests were raised (Fang et al., 2020; Drame et al., 2020). This study suggests that patients who were tested for SARS-CoV-2 but received a negative PCR test may have been infected with SARS-CoV-2. While we do not know how many of the F-N patients were symptomatic, or asymptomatic, our data show that prior to filtering, 11% of PCR-negative samples showed evidence of SARS-CoV-2 infection using RNA sequencing (Figure S1A). Earlier tests relied on a single SARS-CoV-2 gene for identification, whereas later tests used multiple genes. However, even multi-gene PCR tests are vulnerable to mutations resulting in suboptimal amplification of the target amplicon (such as the S-gene drop out observed in some tests (Volz et al., 2021)). Based on calculations of true positive rates taking sequencing to be the true value, we calculate the accuracy of PCR to be 90% (specificity 97% and sensitivity 79%) (Figure S1C) (Trevethan, 2017); this suggests that PCR is better at detecting if a person does not have SARS-CoV-2 infection. This compares close to a recent review (Zitek, 2020) of nasal-pharyngeal RT-PCR tests (reporting a specificity and sensitivity of 98.8% and 78.2%, respectively based on data in (Wang et al., 2020)). Conversely, if we consider PCR to be the true standard, then sequencing in this study had a sensitivity of 97% and specificity of 87%. Therefore, sequencing could be deemed a better test for detecting infected people, but more costly and too slow for widespread use. It is not clear whether this analysis extends to other PCR assays but warrants further investigation. Collectively, these data suggest the number of reported COVID-19 cases is likely lower than the actual number of individuals who have been infected with the virus.

### Limitations of the study

Our analysis suggests that over 10% of patients with negative PCR results may have been infected with SARS-CoV-2. Clearly, the impact of this estimate on positivity rates would depend on whether the testing was performed as a clinical assay or for surveillance. Furthermore, it is not clear whether people tested were symptomatic, at what stage of the infection they were at, severity, or their outcome. All these confounding factors may affect the expression of genes and may account for the high variability in expression profiles we observe (Figure 3) and the challenge of predicting COVID status based on gene expression alone (Figure S2). Even when we compare control (PCR negative) samples, we observe high variability in the data (see Figure S2). Nasal-pharyngeal transcriptomes are less studied than other tissues, such as blood, hence the natural variation of this tissue sample is less well understood. Regardless of absolute gene expression, it was clear that biologically related genes were regulated in the disease (Figure 5), and sequencing enabled us to identify those signatures, as well as reads from across the SARS-CoV-2 genome. Taken together, this study shows over 10 % of PCR-negative samples were likely positive for SARS-CoV-2 suggesting estimate of prevalence of COVID-19 may be underestimated worldwide. More large-scale clinical data to validate these approaches are recommended in future work.

## STAR★Methods

### Key resources table

**Table:** 

REAGENT or RESOURCE	SOURCE	IDENTIFIER
**Deposited data**		

RNA Seq Data	dbGAP	phs002258.v1.p1
	CRAN	V4.0.3 for Windows

**Software and algorithms**		

Bash/PBS scripts for aligning data	All original code has been deposited at Github and is publicly available as of the date of	https://github.com/asaravia-butler/COV-IRT/blob/main/RNAseq/Raw_to_Aligned_Data_Pipeline.md.
R scripts for analysis	All original code is available in this paper’s supplemental information	https://github.com/rob-meller/iSCIENCE-COVIDpaper
R (v 4.0.3)		https://www.r-project.org/
HTStream		https://s4hts.github.io/HTStream/
STAR v	Dobin et al., 2013	https://github.com/alexdobin/STAR
Samtools v	Li et al., 2009	www.htslib.org
Picard Tools		https://broadinstitute.github.io/picard/
Limma	Ritchie et al. 2015	https://bioconductor.org/packages/release/bioc/html/limma.html
Enhanced volcano		(https://github.com/kevinblighe/EnhancedVolcano
TopGO		https://bioconductor.org/packages/release/bioc/html/topGO.html
FSGEA	Korotkevich et al. 2019	https://bioconductor.org/packages/release/bioc/html/fgsea.html
MuSIC	Wang et al. 2019	https://xuranw.github.io/MuSiC/articles/MuSiC.html

### Resource availability

#### Lead contact

Further information and requests for resources and reagents should be directed to and will be fulfilled by the lead contact, Robert Meller rmeller@msm.edu.

#### Materials availability

This study did not generate new unique reagents.

#### Data and code availability

The data for this analysis was deposited in the database of Genotypes and Phenotypes dbGAP (accession #38851 and dbGAP: phs002258.v1.phs). The data for analysis in this project (count matrix and traitdata) are available at https://github.com/rob-meller/iSCIENCE-COVIDpaper.
Code for aligning Fastq files is available at https://github.com/asaravia-butler/COV-IRT/blob/main/RNAseq/Raw_to_Aligned_Data_Pipeline.md. Additional code for analysis of samples using R is provided at https://github.com/rob-meller/iSCIENCE-COVIDpaper. Any additional information required to reanalyze the data reported in this paper is available from the lead contact upon request.

### Experimental model and subject details

Experimental details for data generation was reported in Butler et al., (2021).

#### Data collection

Samples were collected and processed through the Weill Cornell Medicine Institutional Review Board (IRB) Protocol 19-11021069. Dates of collected data for SARS-CoV-2 suspected patients was extracted from the electronic health records at NYP-CUIMC. We used data collected starting on March 10th, 2020 through April 20th, 2020. We applied this total RNA-seq platform to 732 clinical samples, including 669 confirmed or suspected COVID-19 cases at New York-Presbyterian Hospital-Weill Cornell Medical Center (NYPH-WCMC). Prior to analysis, duplicate samples, environmental samples, and seq control samples were removed. Only samples with over 2 million human genome aligned reads were included in the expression analysis (Figure S1B). Raw data are available at the database of Genotypes and Phenotypes dbGAP (accession #38851 and dbGAP: phs002258.v1.phs).

Patient specimens were collected with patients’ consent at New York-Presbyterian Hospital-Weill Cornell Medical Center (NYPH-WCMC) and then processed for qRT-PCR. Nasopharyngeal (NP) swab specimens were collected using the BD Universal Viral Transport Media system (Becton, Dickinson and Company, Franklin Lakes, NJ) from symptomatic patients. Sex was not a determining factor (nor recorded) since it was the first wave of the pandemic and we took all samples that came to the hospital.

#### Human nasopharyngeal swab sample collection for RNA-seq analysis

Patient specimens were processed as described in Butler et al. (2021). Briefly, nasopharyngeal swabs were collected using the BD Universal Viral Transport Media system (Becton, Dickinson and Company, Franklin Lakes, NJ) from symptomatic patients. Total Nucleic Acid (TNA) was extracted from using automated nucleic acid extraction on the QIAsymphony and the DSP Virus/Pathogen Mini Kit (Qiagen).

#### PCR diagnosis of SARS-CoV-2

The PCR methodology was previously published (Butler et al., 2021). Briefly, total viral RNA was extracted from deactivated samples using automated nucleic acid extraction on the QIAsymphony and the DSP Virus/Pathogen Mini Kit (QIAGEN). One step reverse transcription to cDNA (using random hexamer primers) and real-time PCR (RT-PCR) amplification of viral targets, E (envelope) and S (spike) genes and internal control, was performed using the Rotor-Gene Q thermocycler (QIAGEN).

Clinical samples were extracted as described above and then tested with qRT-PCR using primers for the E (envelope) gene, which detects all members of the lineage B of the beta-CoVs, including all SARS, SARS-like, and SARS-related viruses, and a second primer set for the S (spike) gene, which specifically detects the SARS-CoV-2 virus. The reaction also contains an internal control that served as an extraction control and a control for PCR inhibition.

Samples were annotated using qRT-PCR cycle threshold (Ct) value for SARS-CoV-2 primers. Subjects with Ct less than or equal to 18 were assigned “high viral load” label, Ct between 18 and 24 were assigned “medium viral load” and Ct between 24 and 40 were assigned “low viral load” classes, with anything above Ct of 40 classified as negative.

#### RNA-seq of nasopharyngeal swab COVID-19 patient samples

RNA isolation and library preparation is fully described in Butler, et al. (Butler et al., 2021). Briefly, library preparation on all the nasopharyngeal swab samples’ total nucleic acid (TNA) were treated with DNAse 1 (Zymo Research, Catalog # E1010). Post-DNAse digested samples were then put into the NEBNext rRNA depletion v2 (Human/Mouse/Rat), Ultra II Directional RNA (10 ng), and Unique Dual Index Primer Pairs were used following the vendor protocols from New England Biolabs. Kits were supplied from a single manufacturer lot. Completed libraries were quantified by Qubit or equivalent and run on a Bioanalyzer or equivalent for size determination. Libraries were pooled and sent to the WCM Genomics Core or HudsonAlpha for final quantification by Qubit fluorometer (ThermoFisher Scientific), TapeStation 2,200 (Agilent), and qRT-PCR using the Kapa Biosystems Illumina library quantification kit.

#### Data alignment

Raw RNA sequence data from the nasopharyngeal swab samples were uploaded to the NASA HECC supercomputing system (NASA Ames Research Center, Mountain View, CA) and processed as described in https://github.com/asaravia-butler/COV-IRT/blob/main/RNAseq/Raw_to_Aligned_Data_Pipeline.md. First, adapters and low-quality data were trimmed with Trimmomatic (v0.39) (Bolger et al., 2014). Raw and trimmed read quality were evaluated with FastQC (v0.11.9) (https://www.bioinformatics.babraham.ac.uk/projects/fastqc/), and MultiQC (v1.9) (Ewels et al., 2016) was used to generate MultiQC reports. Trimmed reads were split according to sequencing flow cell and lane using gdc-fastq-splitter (v1.0.0) (https://github.com/kmhernan/gdc-fastq-splitter) for subsequent batch effect evaluation, and ribosomal RNA (rRNA) was removed using HTStream (v1.3.2) (https://github.com/s4hts/HTStream). *Homo sapiens* and SARS-CoV-2 STAR reference were built using STAR (v2.7.3a) (Dobin et al., 2013) with Ensembl release 100 human genome version GRCh38 (Homo_sapiens.GRCh38.dna.primary_assembly.fa) concatenated with the SARS-CoV-2 Wuhan-Hu-1 reference genome ASM985889v3 (Sars_cov_2.ASM985889v3.dna.toplevel.fa), and the following Ensembl gtf annotation file: Homo_sapiens.GRCh38.100.gtf concatenated with Sars_cov_2.ASM985889v3.101.gtf. rRNA-depleted trimmed reads were aligned to the *Homo sapiens*, and SARS-CoV-2 STAR reference with STAR twopassMode and quantitated with STAR GeneCounts feature (v2.7.3a) (Dobin et al., 2013).STAR counts from reads aligned to the second, reverse strand from each sample were merged into a combined data matrix for subsequent analysis using R (v3.6.0).

### Quantification and statistical analysis

#### Differential gene expression

Count data and trait data were analyzed using LIMMA in R (V4.0.2) (Ritchie et al., 2015). We excluded rRNA reads from the count data frame (560 gene ids) to reduce the impact of variable rRNA depletion (see above). We first matched samples to the phenotype data, and then removed duplicated samples. Samples were excluded from analysis if they had fewer than 2 million transcriptome aligned counts (564 unique samples) (Figure S1). The dataframe was then saved as a phenotype data frame and a counts matrix. Only genes whose differential expression passed threshold were included in the analysis (approx. 10 counts/ gene/ sample). The design matrix included PCR call, and sequencing batch data extracted from the phenotypic data frame. (∼ 0 + SequencingBatch + PCR_SEQ), where PCR_SEQ was a determination of viral load. Viral load status was used as contrasts for differential gene expression calculations (High, Med, Low, F-N, None or viral : see script for details). Differential gene expression was calculated using Limma-voom, with a Bayesian correction (Ritchie et al., 2015). Contrasts were calculated to be significantly different with an absolute fold change +/− 1.2 fold and adjusted p value <0.05 (Benjamini Hochberg correction). MA plots were generated in Limma. Resultant data tables were used for subsequent analysis or plots (see Data S1). Volcano plots were generated using enhanced_volcano from lists of differentially expressed genes (https://github.com/kevinblighe/EnhancedVolcano).

#### Gene ontology analysis

Tables of 1.2 fold gene changes, adj p values (<0.05) were analyzed using topGO (Data S2). The t-values from the entire data set was used for pathway analysis utilizing fast Gene Set Enrichment Analysis (fGSEA) (Korotkevich et al., 2019). Pathway analysis was performed comparing different viral loads to the controls and the ranked list of genes were defined by the t-score statistics (Data S3). The statistical significance was determined by 1,000 permutations of the gene sets (Subramanian et al., 2005). Resultant pathways were illustrated using Venn diagrams and lollipop figures (Figures 4 and 5).

#### PCA and tSNE analysis

Expression data were converted from counts to cpm and filtered for differentially expressed genes (adj p < 0.05 (FDR) ± 1.2 fold change vs negative control samples). Data from High, Med, Low and F-N, and Neg subjects were then filtered for top 100, 10 and 5 differentially expressed genes (based on adjusted p values), and expression values were used for principal component analysis and tSNE analysis using the inbuilt prcomp function and tsne packages in R (see script). Data were colored based on PCR status/ viral load. Data are from the 483 samples with greater than 2 million aligned human reads. Random forest and neural network predictions were made on the top 100, 10 and 5 differentially expressed genes, using the randomForest and neuralnet packages. The data were split into training and testing datasets, and the goal was to correctly predict the viral load status (High, Med, low, Negative) of the F-N data (see script).

## Supplemental information

Document S1. Figures S1–S5

Data S1. Spreadsheet of gene expression values used in this analysis related to Figure 3

Data S2. Spreadsheet of GoBP terms for different datasets (separated by viral load) related to Figure 4 and 5

Data S3. Spreadsheet of FSGEA results from gene expression values (separated by viral load)
