# Supplementary material for: Host transcriptional responses in nasal swabs identify potential SARS-CoV-2 infection in PCR negative patients
Source: iScience. 2022 Oct 7;25(11):105310. doi: 10.1016/j.isci.2022.105310 (PMC9540688; doi:10.1016/j.isci.2022.105310)
Supplement: Document S1. Figures S1–S5 [file mmc1.pdf]

iScience, Volume *25*

Supplemental information

Host transcriptional responses in nasal swabs identify potential SARS-CoV-2 infection in PCR negative patients

Amanda M. Saravia-Butler, Jonathan C. Schisler, Deanne Taylor, Afshin Beheshti, Dan Butler, Cem Meydan, Jonathon Foox, Kyle Hernandez, Chris Mozsary, Christopher E. Mason, and Robert Meller

Supplementary Figure 1

A

PCR_SEQ	All Data	Remove duplicates	Filter low reads
PCR+ve No Seq	16	10	6
High	62	61	52
Low	42	39	33
Med	101	92	81
Neg	380	343	275
NF-N	49	46	42
Viral_Neg	73	70	66
Viral_Pos	9	9	9
**Total**	**732**	**670**	**564**
%	11%	12%	13%

B

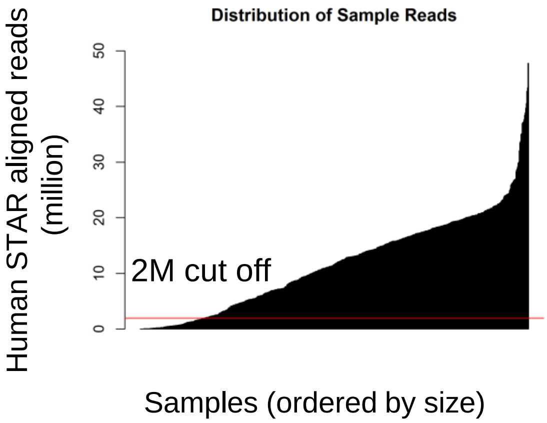

C

		Seq (Actual))	
		+ve	-ve
**PCR**	+ve	166	6
	-ve	42	275

**TP**	166	**FP**	6
**TN**	275	**FN**	42

**Accuracy**	TP + TN / TP + TN + FP + FN	(166+275)/(166+275+6+42)	0.90184
**Misclassification**	FP + FN / TP + TN + FP + FN	(6+42)/(166+275+6+42)	0.09816
**Precision**	TP / TP + FP	166/(166+6)	0.96512
**Sensitivity**	TP / TP + FN	166/(166+42)	0.79808
**Specificity**	TN / TN + FP	275/(275+6)	0.97865

Supplementary Figure 1 related to Figure1. A. Table containing the number of samples in each PCR and sequencing type after each filtering step used in this analysis. Total indicates the total number of samples after each filtering step and % denotes the % of PCR negative samples that showed SARS-CoV-2 presence based on sequencing data. B. Histogram of the number of aligned human reads in each sample, showing effect of removing samples using a 2 million minimum read cut off. C. Calculation of accuracy, misclassification, precision, sensitivity, and specificity taking sequencing as the true value (TP= true positive, TN= True Negative, FP= false Positive, FN = false negative; +ve = positive, -ve = negative).

Supplementary Figure 2

Plate ID	Seq_Neg	High	Low	Med	Neq	F-N	Viral_Neg	Viral_Pos	Total
P1	3	8	10	15	11		3		50
P10		12	9	17	27	8	4	2	79
P11		6	3	7	19	6	4	1	46
P12		5	3	13	16	10	4	3	54
P2		1	1	3	43	3	9		60
P3	3	2	1	1	18		2		27
P4		3		3	19	2	4	1	32
P5		1		2	18	2	2		25
P6		5		4	31	4	7	1	52
P7		4		9	24	1	6		44
P8		2	3	2	24	3	8		42
P9		3	3	5	25	3	13	1	53
**Total**	**6**	**52**	**33**	**81**	**275**	**42**	**66**	**9**	**564**

Supplementary Figure 2 relating the Figure 1. Table showing break down of numbers of samples sequenced by sequencing plate batch.

Supplementary Figure 3

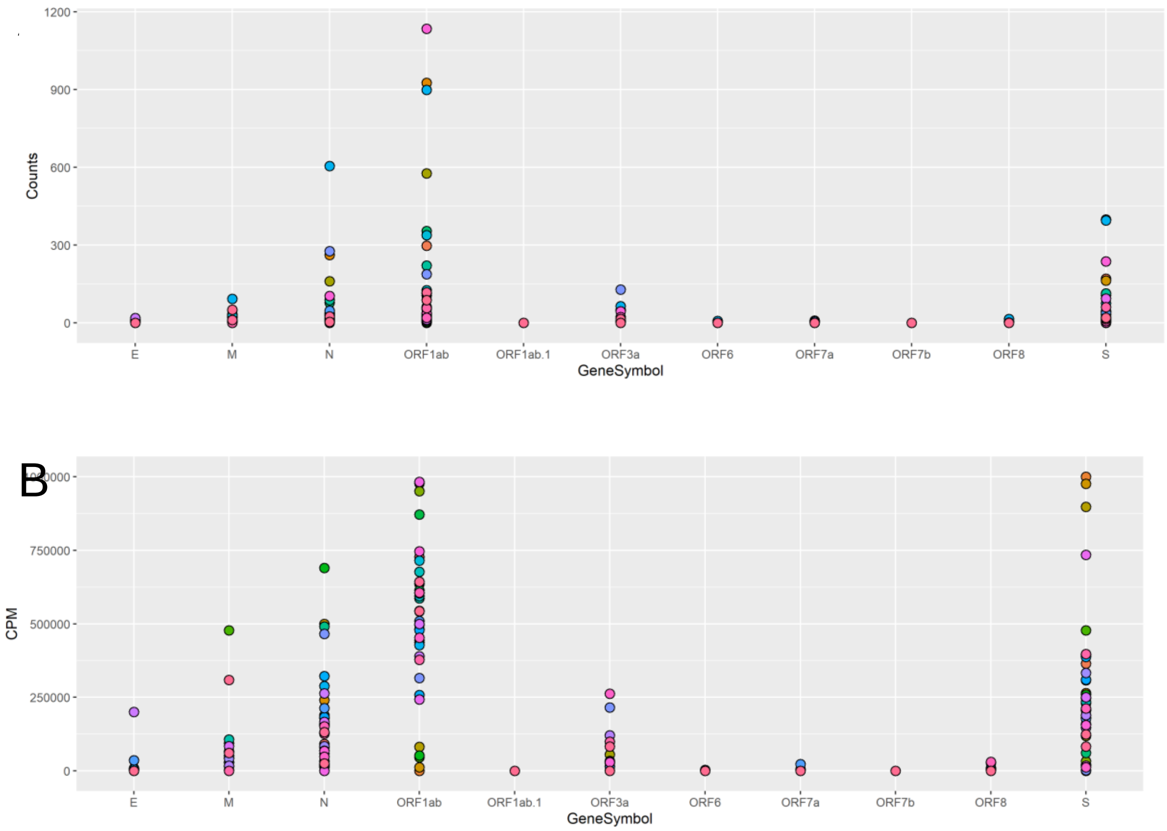

Supplementary Figure 3 Related to Fig 2. Levels of SARS-CoV-2 aligned reads by genomic location as with and without library size normalization. A. Total read counts per sample as determined by gene alignment. B Read counts were converted to CPM and plotted for each PCR negative sequencing positive sample.

Supplementary Figure 4

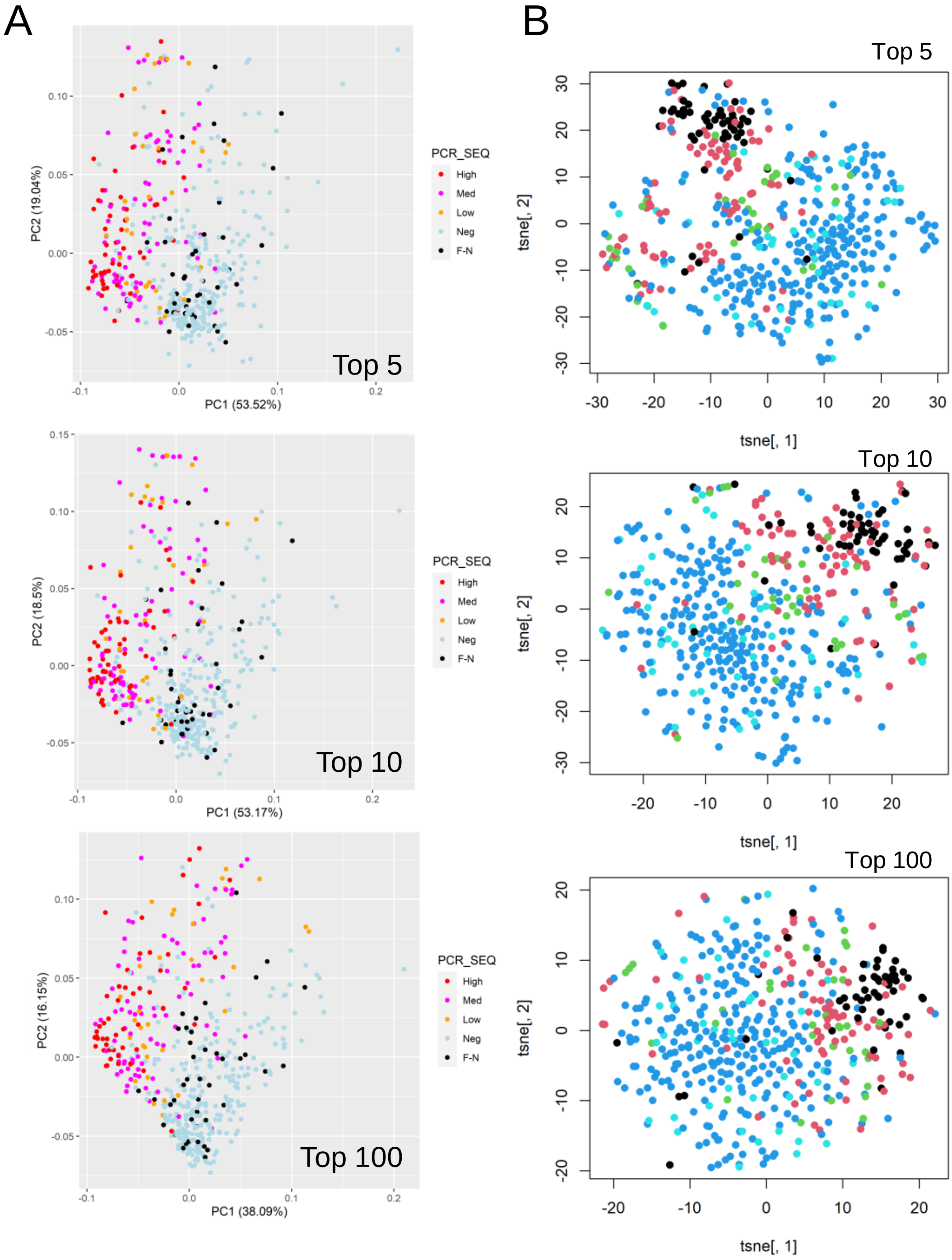

Supplementary Figure 4 related to Fig 3. A. PCA and B. tSNE plots of the top 5, 10 and 100 differentially expressed genes. Data were filtered for differentially expressed genes (adj p<0.05 (FDR) ± 1.2 fold change vs negative control samples). Data from High, Med, Low and F-N, versus Neg subjects (viral load status) were then filtered for top 100, 10, and 5 differentially expressed genes, and expression values were used for principal component analysis and tSNE analysis (see script). Data were colored based on viral load status. Data are from the 483 samples with greater than 2 million aligned human reads. These data show the high degree of variance in the uncorrected data.

Supplementary Figure 5

	Df	Sum Sq	Mean Sq	F value	Pr(>F)	
Viral Load	4	0	0	0	1	
Cell_Type	4	70.2	17.549	616.013	<2e-16	***
Viral Load:Cell_Type	16	0.72	0.045	1.581	0.0657	.
Residuals	2390	68.09	0.028			

Signif. codes: 0 ‘***’ 0.001 ‘**’ 0.01 ‘*’ 0.05 ‘.’ 0.1 ‘ ’ 1

	diff	lwr	upr	p adj.
Med-High	-0.0003	-0.0369	0.03636	1
Low-High	-0.0002	-0.046	0.04571	1
Neg-High	-0.0002	-0.0314	0.03096	1
F-N-High	2.3E-06	-0.0427	0.04275	1
Low-Med	0.0001	-0.0425	0.04266	1
Neg-Med	5.4E-05	-0.026	0.0261	1
F-N-Med	0.00026	-0.0389	0.03944	1
Neg-Low	-5E-05	-0.038	0.03791	1
F-N-Low	0.00016	-0.0478	0.04809	1
F-N-Neg	0.0002	-0.0339	0.03434	1

Supplementary Figure 5 related to Fig 6D. ANOVA results for cell proportion data.
